# Ohio

**Published:** 1897-03

**Authors:** 


### CONTROL WORK.

*Ohio.* The *Breeders' Gazette* quotes two of Ohio's representatives in the medico-sanitary world as follows: At the last meeting of the Ohio Agriculturists Dr. N. S. Kinsman, of the State Cattle Commission and also of the Columbus Board of Health, had a paper on "Tuberculosis." He claimed that not only the milk but the meat of animals affected with tuberculosis is unfit for food; that the disease is more to be dreaded than cholera, diphtheria, and typhoid fever combined. He advocated a State commission for testing all milch-cows with tuberculin and the slaughter of all animals that show reaction, and meat-inspection and compulsory slaughter at abattoirs centrally located. Dr. D. S. White, veterinarian of the State University, followed, and showed that the danger from the use of milk and meat is not so great as claimed by some. He held that tuberculin is not reliable. It has value, but not sufficient to warrant the wholesale slaughter and waste its advocates encouraged. The fact that of the calves of 75,000 cows tested not one calf showed tuberculosis is enough to show that there is little danger of milk inducing the disease.

